# Usability evaluation of a glove-type wearable device for efficient biometric collection during triage

**DOI:** 10.1038/s41598-024-60818-9

**Published:** 2024-04-30

**Authors:** Masayoshi Shinozaki, Daiki Saito, Keisuke Tomita, Taka-aki Nakada, Yukihiro Nomura, Toshiya Nakaguchi

**Affiliations:** 1 Department of Medical Engineering, Center for Frontier Medical Engineering, Graduate School of Science and Engineering, Chiba University, 1-33, Yayoicho, Inage-ku, Chiba-shi, Chiba, 263-8522 Japan; 2 Department of Emergency and Critical Care Medicine, Chiba University Graduate School of Medicine, 1-8-1, Inohana, Chuo-ku, Chiba-shi, Chiba, 260-8677 Japan; 3 Center for Frontier Medical Engineering, Chiba University, 1-33, Yayoicho, Inage-ku, Chiba-shi, Chiba, 263-8522 Japan

**Keywords:** Health services, Public health, Health care, Engineering, Biomedical engineering, Electrical and electronic engineering

## Abstract

To efficiently allocate medical resources at disaster sites, medical workers perform triage to prioritize medical treatments based on the severity of the wounded or sick. In such instances, evaluators often assess the severity status of the wounded or sick quickly, but their measurements are qualitative and rely on experience. Therefore, we developed a wearable device called Medic Hand in this study to extend the functionality of a medical worker’s hand so as to measure multiple biometric indicators simultaneously without increasing the number of medical devices to be carried. Medic Hand was developed to quantitatively and efficiently evaluate "perfusion" during triage. Speed is essential during triage at disaster sites, where time and effort are often spared to attach medical devices to patients, so the use of Medic Hand as a biometric measurement device is more efficient for collecting biometric information. For Medic Hand to be handy during disasters, it is essential to understand and improve upon factors that facilitate its public acceptance. To this end, this paper reports on the usability evaluation of Medic Hand through a questionnaire survey of nonmedical workers.

## Introduction

Injured and ill persons need immediate medical attention, and their physical conditions need to be recorded for continuous evaluation. Because of the usual imbalance between injured and ill patients and medical resources at disaster sites, efficient allocation of medical resources is essential^1–3^.

**Figure 1:**
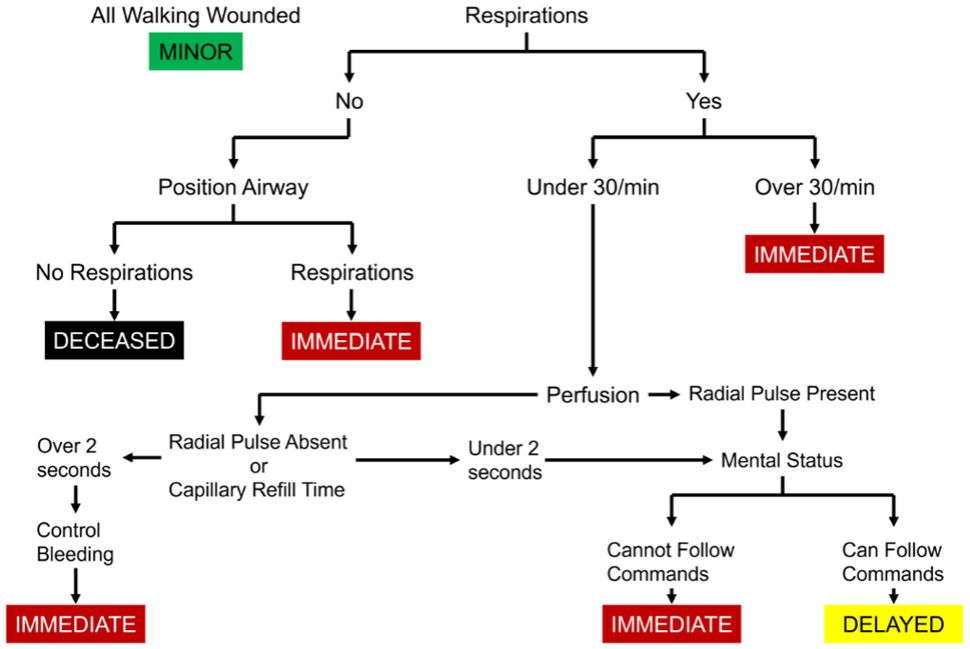
Simple triage and rapid treatment (START) triage method^4^.

To efficiently allocate medical resources at disaster sites, medical workers perform triage to prioritize medical treatment based on the severity of the wounded or sick. Triage at the site of a disaster or accident is performed according to a guideline called the simple triage and rapid treatment (START) triage method^4^. The START triage method evaluates the patient’s condition based on four indicators: ambulation, respiration, perfusion, and mental status (Fig. 1).

According to the results of these four indicators, the wounded and sick are classified into four color groups that indicate their treatment priority:

- GREEN: Minor injuries that do not require immediate medical attention.
- YELLOW: Even seriously injured or wounded can delay treatment for a short period.
- RED: Seriously injured person who needs immediate medical attention.
- BLACK: Injured or ill persons who are challenging to resuscitate and cannot be medically treated.

Evaluators are required to measure these four indicators quickly, but the measurements are qualitative and rely on experience. In disaster medicine, inexperienced residents may become triage providers to the detriment of both the provider and injured or sick patient^1^.

To solve the problem of triage, Dong et al. developed the eTRIAGE®^5–7^. The eTRIAGE® provides users with relevant factors for patients’ symptoms from the Canadian Triage and Acuity Scale (CTAS) database to assist in appropriate triage scoring. Several research groups developed triage systems that use data collected during triage (such as demographics, vital-signs, chief complaint, mode of arrival, and medical history) to predict patient outcomes through machine learning^8–10^. Rivero-García et al.^11^ proposed a complete system using smartphones and near-field communication (NFC) tags to electronically record triage results and classify victims in emergencies according to their severity while simultaneously providing their location and best route to reach them. The widespread use of such electronic triage systems has been shown to reduce the responsibilities of inexperienced residents and contribute to more efficient triage^12,13^. However, the data used in these electronic triage systems are diverse, including textual, qualitative, and quantitative data, making data collection more efficient a challenge. To improve the efficiency of data, especially vital sign collection, Niswar et al.^14^ proposed electronic triage using wearable medical devices that can constantly monitor the health status (respiratory rate, pulse rate, and arterial oxygen saturation) of the injured; a decision system that monitors the vital signs of the injured then classifies them into three levels of severity, namely “IMMEDIATE,” “DELAYED,” and “MINOR” illness. However, the electronic triage proposed by Niswar et al. requires that a vital-signs monitor to be attached to the patient. The number of injured and sick persons at a disaster site is challenging to predict, and this system is unhelpful if there are an insufficient number of monitors.

Therefore, we developed a wearable device called Medic Hand that extends the functionality of a medical worker’s hands and can be used to measure multiple biometric indicators simultaneously without increasing the number of medical devices to be carried. Since the number of medical workers available at a disaster site is more predictable than the number of casualties, it is possible to provide as many Medic Hands as needed. Speed is essential during triage at disaster sites, where even time and effort are often spared to attach medical devices to patients, so using Medic Hand as a biometric measurement device is more efficient for collecting biometric information. While smart wearable devices such as the Medic Hand have much potential for assisting citizens during a disaster, the use of such devices in a disaster is still a new concept to the general public, and many people may resist the unfamiliar technology^15^. Therefore, to ensure that these devices are handy during disasters, it is essential to understand and improve upon the factors that facilitate public acceptance. To this end, the present work reports on the usability evaluation of Medic Hand via a questionnaire survey of nonmedical workers.

## Materials and methods

### Wearable device to measure capillary refill time (CRT)

**Figure 2:**
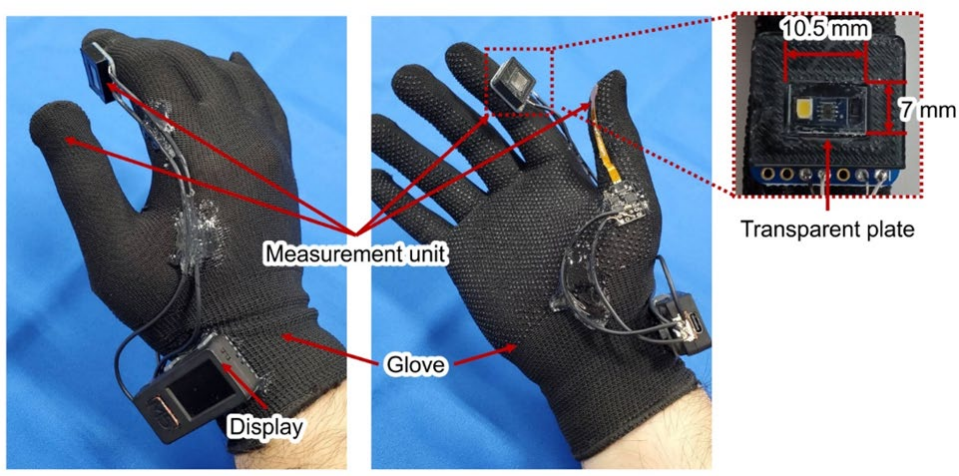
Prototype wearable CRT measurement device: Medic Hand.

**Figure 3:**
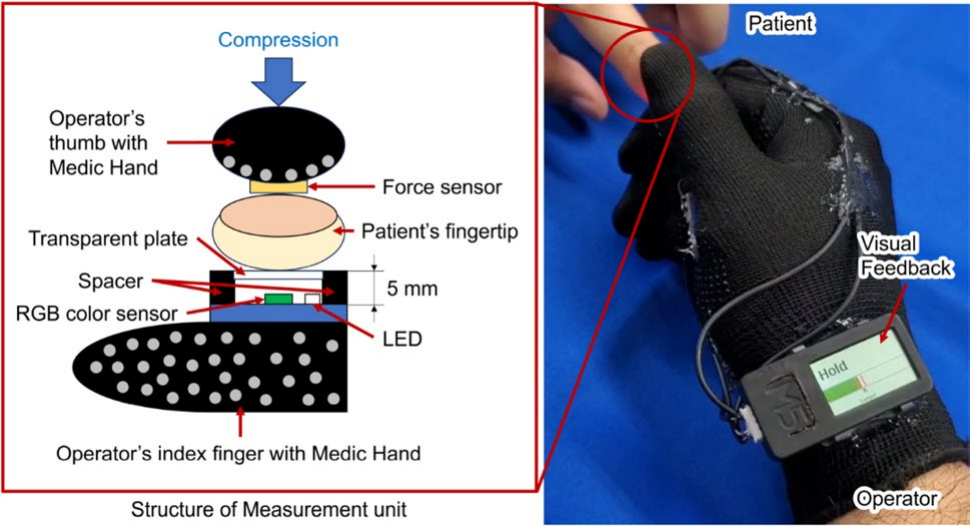
CRT measurement with Medic Hand.

**Figure 4:**
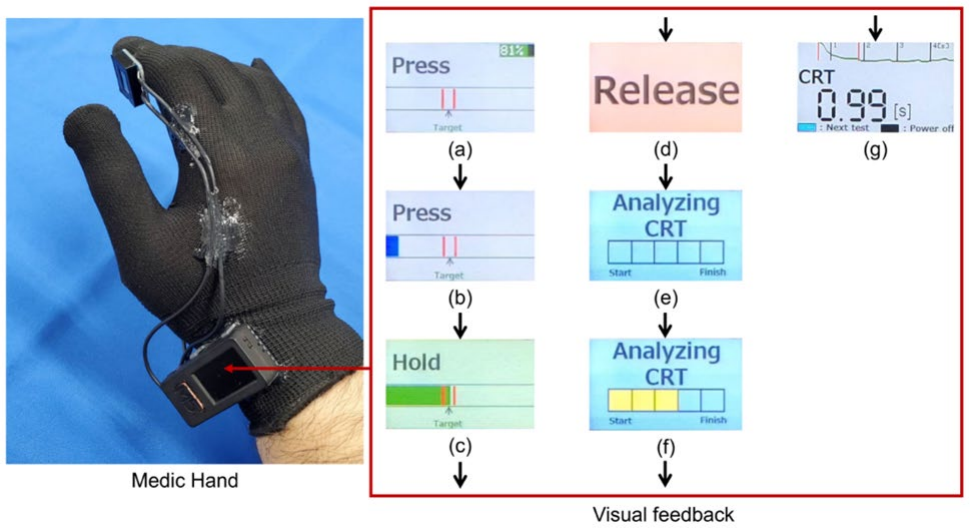
Visual feedback function for measuring CRT using Medic Hand.

The prototype of Medic Hand is shown in Fig. 2; it measures the finger pulp color and provides visual feedback on the compression strength and time^16^. The measurement unit (Fig. 2) comprises a force sensor (Single Tact 8-mm Diameter Sensor 10 N/2.2 lb., Medical Tactile Inc., USA), a color sensor (RGB Color Sensor with IR cut filter, TCS34725, Adafruit Industries, USA), and a spacer with a transparent plate (size: 10.5 mm × 7 mm) that contacts the finger pulp. The spacer is printed using a 3D printer and is used to maintain a constant distance between the measurement unit and finger pulp (Fig. 3). The measured finger pulp color, compression strength, and capillary refill time (CRT) can be stored in an external memory (Micro SD). When measuring the CRT, Medic Hand is attached to the operator’s right hand so that the subject’s fingertip can be pinched through compression and release according to the feedback function (Figs. 3 and 4). The feedback function is in terms of visual feedback displayed on a TFT LCD whose color changes based on the compression force measured by the force sensor.

Figure 4a indicates completion of measurement preparation. The number (%) in the upper-right corner of Fig. 4a indicates the remaining battery power. The black box at the center of the image indicates the compression force applied by the operator. The two red lines at the center of the box indicate the desired compression force range of 5 to 6 N; the target compression force applied by the operator should be between these two red lines. Figure 4b indicates that the compression has started; if the bar indicating the applied compression force is blue, then it indicates that the force applied is less than 3 N. Figure 4c shows maintenance of the compression force, where the display transitions from Fig. 4b when the force applied by the operator increases above 3 N. At this point, the indicator bar and screen background are both green, denoting that the compression force is between 3 and 7 N. The maintenance time of compression is measured from the point at which the display transitions to Fig. 4c. Figure 4d indicates the release of pressure. The visual feedback function maintains Fig. 4c on the display unit for 3 s before transitioning to Fig. 4d. Once the operator stops applying the compression force, the release time of compression is measured until the point the compression force becomes less than 1 N. Figure 4e indicates the process of data storage, and the display transitions from Fig. 4d once the compression has ceased and force drops below 1 N. Figures 4e and f indicate the process of data storage and CRT calculation, and the display transitions from Fig. 4d once the compression has ceased and the force drops below 1 N. The operator must be careful to not remove the fingertip from Medic Hand or cause any vibrations to Medic Hand during measurement. The yellow boxes indicate the elapsed time at the rate of one for each second. The CRT is calculated based on the change in reflected light intensity over 5 s, recorded over the time when the display shows Fig. 4d–f. The average sampling rates of the color and force sensors are 83 Hz each. Figure 4g shows the measured result on the display. The display transitions from Fig. 4f once the CRT calculation is complete. The display unit shows the calculated CRT value and recovery curve for the finger pulp color. The green line indicates the output value of the green channel of the color sensor, and the interval marked with the red lines indicates the CRT. Thus, the visual feedback function ensures that the patient’s fingertip is compressed with a force exceeding 3 N but below 7 N for more than 3 s, with the finger pulp color being measured automatically. In this study, the target compression strength and compression time were set to 5 N and 3 s, respectively^17^. We demonstrate the feasibility of the Medic Hand for measuring CRT by evaluating the agreement, repeatability, and intra-rater reliability of the measurements^18^.

### Medic hand for measuring multiple biometric data

**Figure 5:**
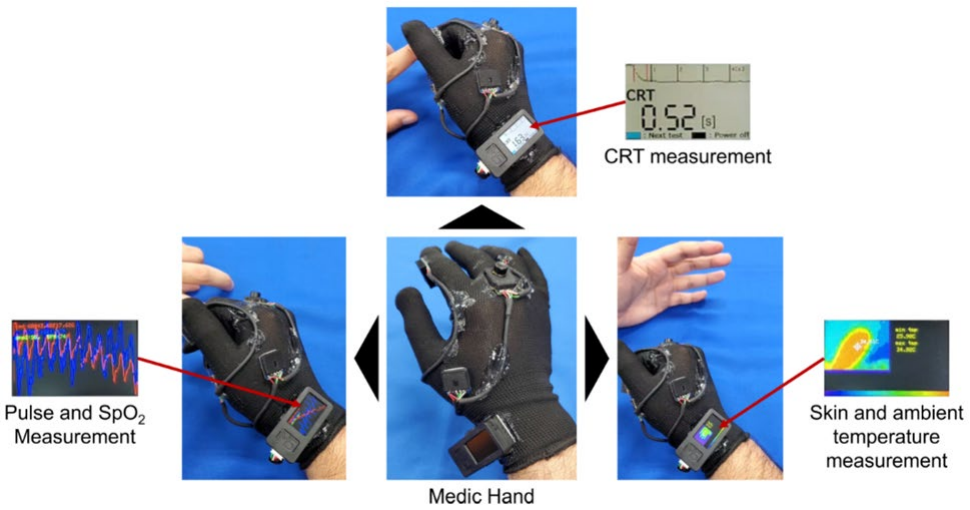
Medic Hand for measuring multiple biometric data during triage.

We developed the multi-biometric Medic Hand (Fig. 5) to measure the CRT, heart rate and oxygen saturation (heart rate monitor, MAXREFDES117#, Analog Devices Inc., USA), skin temperature, and ambient temperature (Thermal Camera Breakout Wide angle 110°, MLX90640, Pimoroni Ltd., UK). Medic Hand measures the ambient temperature with a thermo-camera and can help avoid hazardous environments, such as fires at disaster sites. Medic Hand can also measure circulatory parameters such as the CRT, heart rate, oxygen saturation, and skin temperature efficiently.

CRT measurements using Medic Hand are described in Figs. 3 and 4. The patient’s finger pulp is placed on the color sensor, and a force sensor attached to the wearer’s thumb is used to press and release the patient’s fingertip to measure skin color changes necessary for calculating the CRT (Fig. 5). The pulse rate and oxygen saturation are measured by placing the patient’s finger on a heart rate and oxygen saturation sensor attached to the proximal interphalangeal (PIP) joint of the ring finger (Fig. 5). The skin and ambient temperatures are measured by photographing the object with a thermal camera attached to the metacarpophalangeal (MP) joint of the middle finger (Fig. 5). The user can switch the measurement functions by pressing a button in the following order: CRT, pulse rate and oxygen saturation, and skin and ambient temperature. These show that Medic Hand can help with efficient collection of biometric data in environments where the balance between numbers of medical workers and injured or sick is disrupted, such as disaster sites.

**Figure 6:**
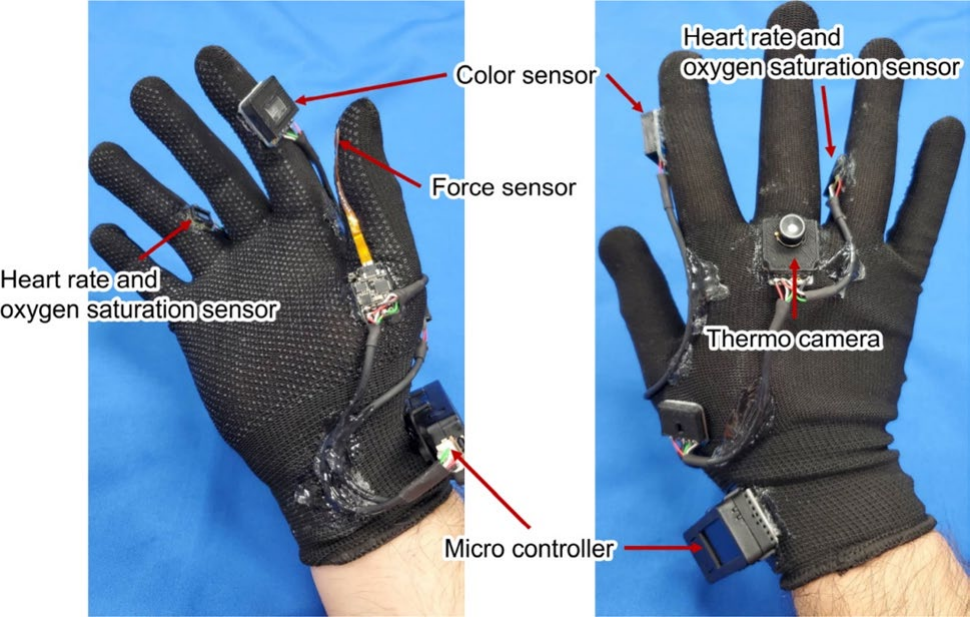
Arrangement of the sensors on Medic Hand.

Each sensor in Medic Hand is attached to the glove using elastic adhesive (Super X AX-041, CEMEDINE Co., Ltd., Japan) and connected to the microcontroller via a flexible robotic cable (Slim Robot Cable, KRT AWG28×4C, KYOWA HARMONET LTD., Japan) (Fig. 6).

### Questionnaire to evaluate the usability of medic hand

Andreoni assessed the reality of usability evaluation for wearable systems^19^. Their results revealed that questionnaires such as the system usability scale (SUS) and user experience questionnaire (UEQ) were used for 70.3% of the usability evaluations of wearable devices. Therefore, we decided to evaluate the usability of Medic Hand using the SUS and UEQ in this study.

### System usability scale (SUS)

The SUS is a 10-item (Table 1), 5-point satisfaction questionnaire designed to measure the subjective satisfaction of the users^20^. The scoring flow is as follows:

**Table 1:** System usability scale (SUS) questionnaire.

Number	Questions
1	I think that I would like to use this system frequently
2	I found the system unnecessarily complex
3	I thought the system was easy to use
4	I think that I would need the support of a technical person to be able to use this system
5	I found that the various functions in this system were well integrated
6	I thought there was too much inconsistency in this system
7	I would imagine that most people would learn to use this system very quickly
8	I found the system very cumbersome to use
9	I felt very confident using the system
10	I needed to learn a lot of things before I could get going with this system

### Scoring of the SUS

1. Scale user responses [numbers 1 (Strongly disagree) to 5 (Strongly agree)] to the range of 0–4 as follows. For odd-numbered questions, subtract 1 from the answer number. For even-numbered questions, subtract the answer number from 5.
2. Add the converted values and multiply by 2.5.

In the SUS evaluations, a score of 68 is a benchmark that can be considered average usability^21^.

### User experience questionnaire (UEQ)

**Figure 7:**
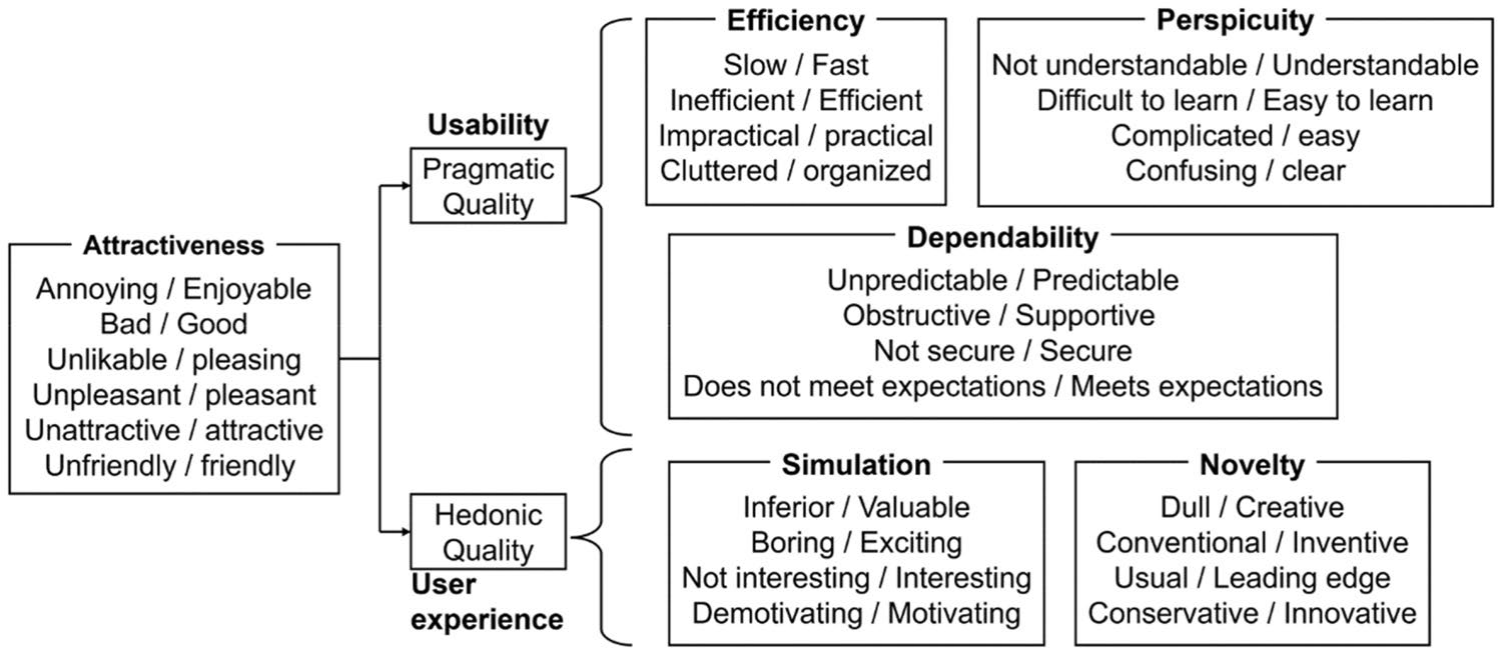
Correspondence between each question and each scale^22^.

The UEQ consists of 26 questions (Table 2) evaluated on six scales: “attractiveness,” “perspicuity,” “efficiency,” “dependability,” “stimulation,” and “novelty^22^. The correspondence between each question and each scale is shown in Fig. 7.

**Table 2:** User experience questionnaire (UEQ) items.

Number	Questions
1	Annoying/enjoyable
2	Not understandable/understandable
3	Creative/dull
4	Easy to learn/difficult to learn
5	Valuable/inferior
6	Boring/exciting
7	Not interesting/interesting
8	Unpredictable/predictable
9	Fast/slow
10	Inventive/conventional
11	Obstructive/supportive
12	Good/bad
13	Complicated/easy
14	Unlikable/pleasing
15	Usual/leading edge
16	Unpleasant/pleasant
17	Secure/not secure
18	Motivating/demotivating
19	Meets expectations/does not meet expectations
20	Inefficient/efficient
21	Clear/confusing
22	Impractical/practical
23	Organized/cluttered
24	Attractive/unattractive
25	Friendly/unfriendly
26	Conservative/innovative

The scoring flow is as follows:

### Scoring of the UEQ

1. Answer each question with a number from 1 to 7.
2. The order of the positive and negative terms for an item is randomized in the questionnaire.
3. Per the dimension, half of the items start with the positive and half with the negative terms.
4. Convert numbers 1–7 to the range of − 3 to 3. Here, + 3 represents the most positive value while − 3 is the most negative value.
5. The mean and confidence interval are calculated for each scale.

The benchmarks used in the evaluation of the UEQ (Table 3) are as presented by Schrepp et al.^22^.

**Table 3:** Benchmarks of the UEQ.

	Att.	Eff.	Per.	Dep.	Sti.	Nov.
Excellent	$\ge 1.75$	$\ge 1.78$	$\ge 1.90$	$\ge 1.65$	$\ge 1.55$	$\ge 1.40$
Good	$\ge 1.52$	$\ge 1.47$	$\ge 1.56$	$\ge 1.48$	$\ge 1.31$	$\ge 1.05$
	$<1.75$	$<1.78$	$<1.90$	$<1.65$	$<1.55$	$<1.40$
Above average	$\ge 1.17$	$\ge 0.98$	$\ge 1.08$	$\ge 1.14$	$\ge 0.99$	$\ge 0.71$
	$<1.52$	$<1.47$	$<1.56$	$<1.48$	$<1.31$	$<1.05$
Below average	$\ge 0.7$	$\ge 0.54$	$\ge 0.64$	$\ge 0.78$	$\ge 0.50$	$\ge 0.30$
	$<1.17$	$<0.98$	$<1.08$	$<1.14$	$<0.99$	$<0.71$
Bad	$<0.7$	$<0.54$	$<0.64$	$<0.78$	$<0.50$	$<0.30$

### Experimental protocol and statistical analysis

We evaluated the usability of Medic Hand for measuring multiple biometric data. This experimental study was approved by the Institutional Review Board of Chiba University Graduate School of Medicine (approval number: 4154). Sixteen nonmedical adults in their 20 s (8 males and 8 females) who majored in medical engineering participated in the study as subjects. For experiments on human subjects, informed consent was obtained from all subjects who participated in the study. We confirmed that all experiments were performed in accordance with relevant guidelines and regulations. The developer explained the background and use of Medic Hand to the evaluators along with demonstrations. The evaluators then measured the CRT, oxygen saturation, pulse rate, skin temperature, and ambient temperature using Medic Hand. After obtaining measurements, the developer provided a questionnaire to the evaluators. At this time, the evaluators were asked to answer the questionnaire by intuition. Once the questionnaires were completed, the developer interviewed the evaluators for the following questions.

Questions to the evaluators:

1. Did you have any fear of wearing this device?
2. Would you want to use such a device in your daily life?
3. What new features would you like to see added to this device for daily use?
4. Do you think the Medic Hand wearable device is new?

The SUS questionnaire was developed by Brooke et al.^20^ and analyzed using Microsoft Office 365 Excel. The UEQ was created by Schrepp et al. and analyzed using the data analysis tools developed by Schrepp et al.^22^.

## Results

### System usability scale (SUS)

**Figure 8:**
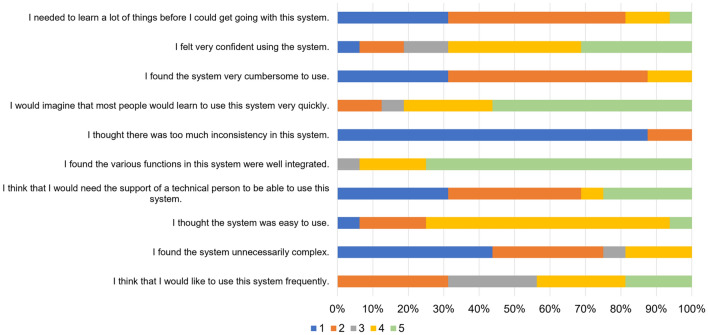
A 100% stacked horizontal bar graph of the evaluators’ responses to the SUS question items. The distribution of colors in each bar means user responses [numbers 1 (Strongly disagree) to 5 (Strongly agree)].

A 100% stacked horizontal bar graph of the evaluators’ responses to the SUS question items is shown in Fig. 8. The distribution of colors in each bar means user responses [numbers 1 (Strongly disagree) to 5 (Strongly agree)]. All responses were positive with the exception of question 10. The mean SUS score of the 16 participants was 74.4 (Table 4), which exceeded the benchmark score of 68, indicating that the proposed Medic Hand has above-mean usability.

**Table 4:** SUS scores of the 16 evaluators.

Evaluators	Sex	SUS score
1	Male	92.5
2	Female	60.0
3	Male	90.0
4	Female	67.5
5	Male	40.0
6	Female	60.0
7	Female	65.0
8	Male	70.0
9	Male	82.5
10	Male	60.0
11	Female	57.5
12	Female	90.0
13	Male	87.5
14	Male	87.5
15	Female	87.5
16	Female	92.5
Mean (Standard deviation)		74.4 (16.3)

### User experience questionnaire (UEQ)

**Figure 9:**
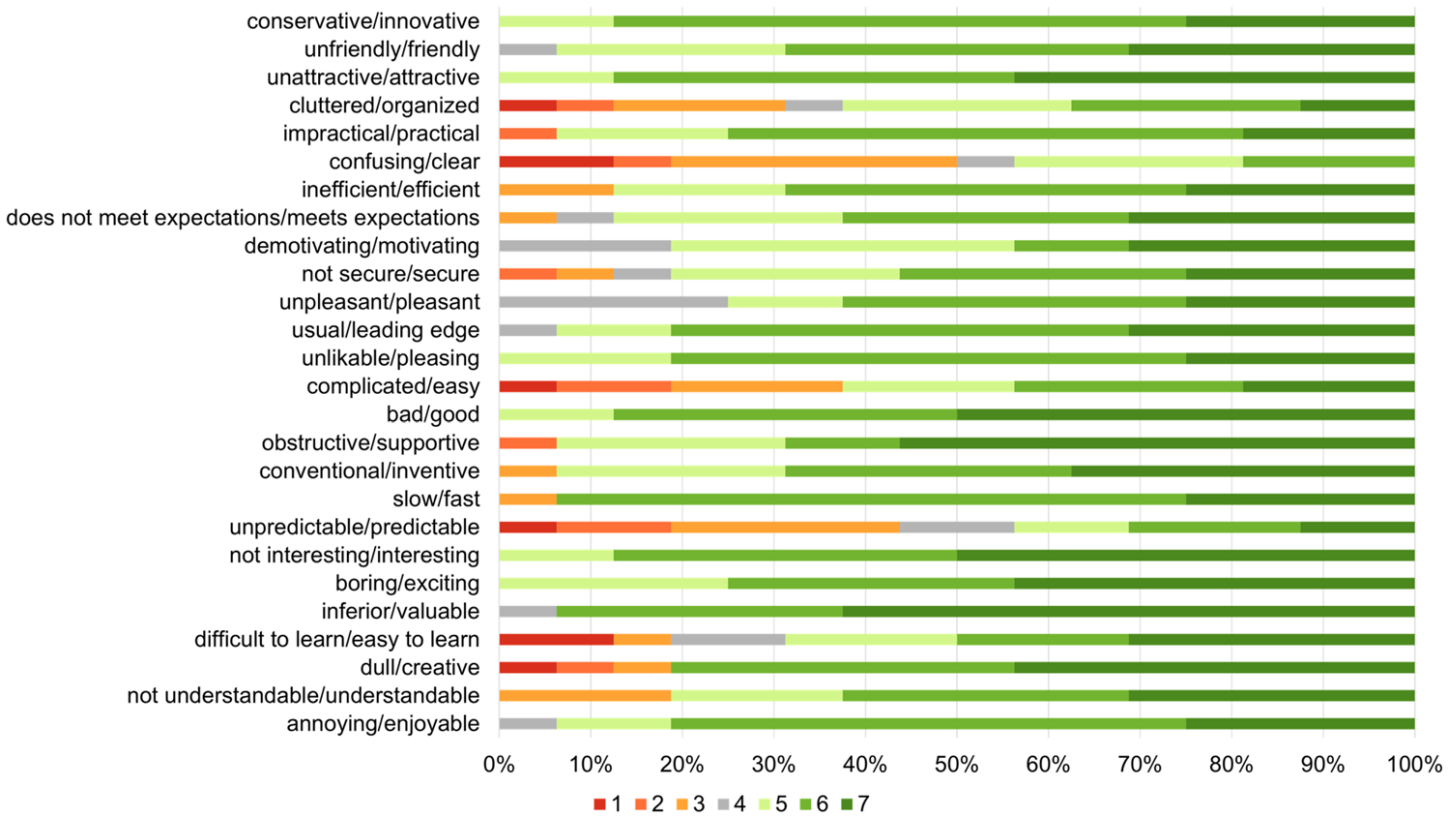
A 100% stacked horizontal bar graph of the evaluators’ responses to the UEQ items. The distribution of colors in each bar means user responses (numbers 1 to 7).

**Figure 10:**
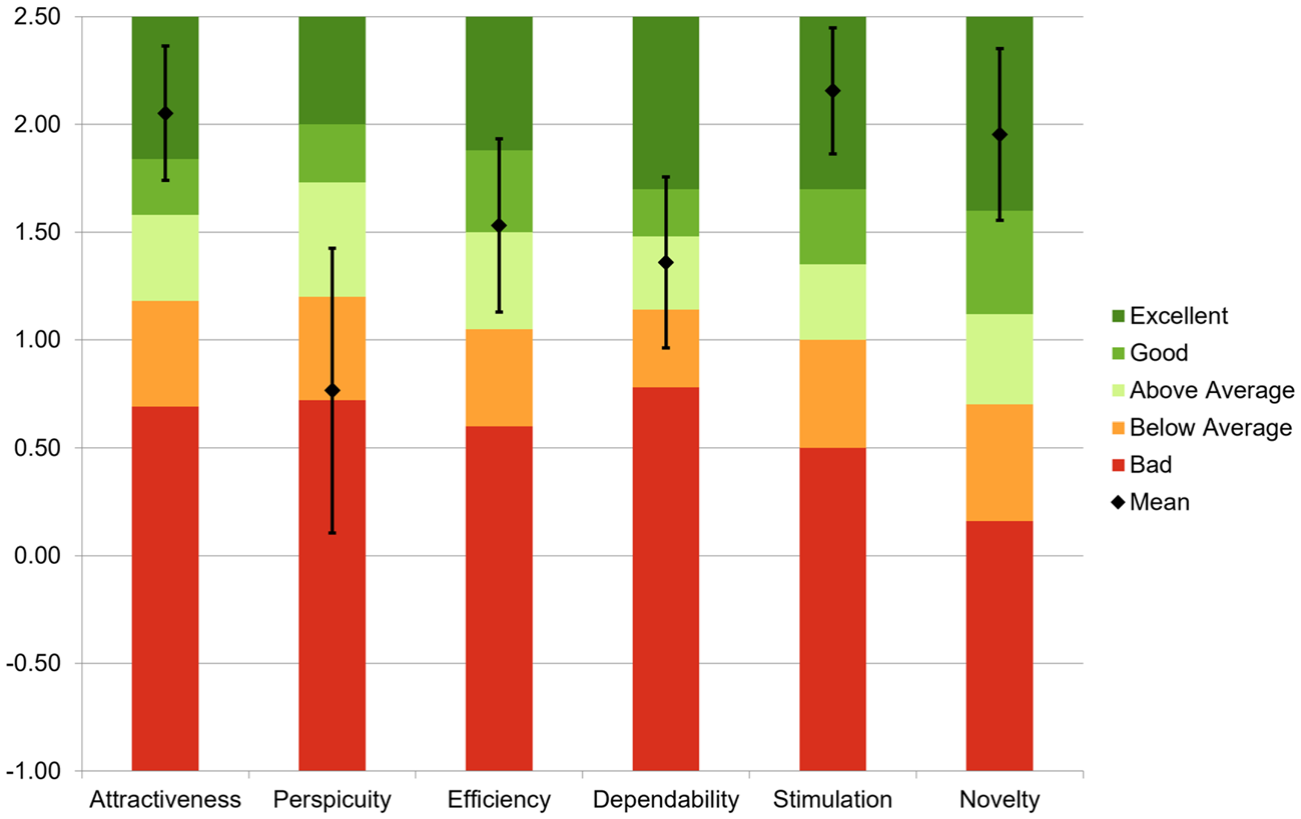
Comparison of means with the benchmarks of Schrepp et al.^22^. Error bars indicate confidence intervals.

A 100% stacked horizontal bar graph of the evaluators’ responses to the UEQ items is shown in Fig. 9. The distribution of colors in each bar means user responses (numbers 1 to 7). All responses were positive with the exception of questions 8 and 21. The mean for each scale for each evaluator is shown in Table 5, and a comparison of the means with the benchmarks of Schrepp et al. is shown in Fig. 10. All scales except “perspicuity” were scored above average. “Attractiveness,” “stimulation,” and “novelty” were rated Excellent on the user experience scale, indicating that the evaluators had a positive impression of Medic Hand.

**Table 5:** Mean and confidence interval (CI) for each scale for each evaluator.

Evaluators	Sex	Att.	Per.	Eff.	Dep.	Sti.	Nov.
1	Male	2.00	1.00	2.00	2.25	2.50	1.00
2	Female	2.67	2.50	2.50	1.75	2.25	1.75
3	Male	2.00	− 1.25	0.75	2.50	1.75	3.00
4	Female	2.00	0.25	2.00	0.75	1.50	2.00
5	Male	3.00	0.50	2.25	2.50	3.00	2.75
6	Female	2.00	− 2.00	0.50	0.50	2.50	3.00
7	Female	1.83	− 0.25	0.75	1.00	2.25	2.75
8	Male	2.33	2.00	1.25	0.75	2.25	2.00
9	Male	1.67	− 0.50	− 0.25	0.75	1.50	2.50
10	Male	0.83	1.75	2.00	0.25	1.50	2.00
11	Female	1.50	2.50	1.50	2.25	2.25	1.25
12	Female	1.33	0.50	1.25	0.75	1.00	0.25
13	Male	1.33	1.00	1.50	1.75	1.75	0.75
14	Male	2.50	1.50	2.00	2.00	2.75	2.00
15	Female	3.00	0.25	1.50	0.25	3.00	1.75
16	Female	2.83	2.50	3.00	1.75	2.75	2.50
Mean		2.05	0.77	1.53	1.36	2.16	1.95
CI		1.74 to 2.36	0.11 to 1.43	1.13 to 1.93	0.96 to 1.76	1.86 to 2.45	1.56 to 2.35

### Interview after questionnaire evaluation

Once the questionnaires were completed, the evaluators were interviewed for the four questions (Experimental Protocol and Statistical Analysis section) mentioned previously (Table 6). “Not applicable” in Table 6 means that no clear answer was received from the evaluator. 13 out of 16 evaluators were afraid to wear the Medic Hand (Question 1). They cited concerns that the exposed wiring and electronic board would be damaged during wearing. 8 out of 16 evaluators indicated that they would not use the Medic Hand for routine biometric measurements (Question 2). They cited as their rationale that, being young and healthy, they were not in the habit of measuring their condition on a daily life. In addition, Thay cited resistance to using products on a daily life that might be damaged. Among the features they would like to see added to the Medic Hand were stress levels, blood pressure, skin condition, axillary temperature, plasma glucose, end-of-measurement notification, tactile sensation, and left-hand use (Question 3). 15 out of 16 evaluators indicated that the Medic Hand was a new device (Question 4). They cited as their rationale that the device has the attraction of a system that can efficiently measure multiple biometric data just by wearing it.

**Table 6:** Results of interviews conducted after the questionnaire evaluation.

Evaluators	Sex	Q1	Q2	Q3	Q4
1	Male	Not applicable	Not applicable	Not applicable	Yes
2	Female	Yes	Yes	Stress levels	Yes
3	Male	Yes	Yes	Not applicable	Yes
4	Female	Not applicable	Not applicable	Not applicable	Yes
5	Male	Yes	No	Blood pressure	Yes
6	Female	Yes	No	Skin condition	Yes
7	Female	Yes	No	Not applicable	Yes
8	Male	Yes	No	Not applicable	Yes
9	Male	Yes	Yes	Blood pressure and axillary temperature	Yes
10	Male	Yes	Not applicable	Blood pressure, axillary temperature, and plasma glucose	Yes
11	Female	Not applicable	No	No	Not applicable
12	Female	Yes	No	No	Yes
13	Male	Yes	Yes	Notification of end of measurement	Yes
14	Male	Yes	Yes	Tactile sensation	Yes
15	Female	Yes	No	Use with left hand	Yes
16	Female	Yes	No	Skin condition and blood pressure	Yes

“Not applicable” means that no clear answer was received from the evaluator.

## Discussion

We evaluated the usability of Medic Hand sixteen nonmedical adults in their 20 s who majored in medical engineering using the SUS and UEQ. The mean SUS score of the evaluators was 74.4, which exceeded the benchmark value of 68, indicating that Medic Hand has above-mean usability. However, 7 out of 16 (male: 2, female: 5) evaluators provided below the benchmark SUS scores, with 62.5% of the female evaluators scoring SUS below the benchmark. Medic Hand was primarily designed to be worn by men, which made it a problematic wearable device for female evaluators to use. In fact, 75% of the male evaluators scored Medic Hand above the benchmark, indicating that its usability was good for men. Therefore, to improve the usability of Medic Hand, it is essential to optimize the design for the wearer’s hand.

In the UEQ evaluations, all scales except “perspicuity” were scored above average. “Attractiveness,” “stimulation,” and “novelty” were rated Excellent on the user experience scale, indicating that the evaluators had a positive impression of Medic Hand. The reason why only “perspicuity” was scored below average was the low evaluation for the “clear/confusing” and “complicated/easy” questions. Medic Hand is simply a cloth glove to which sensors and wiring are attached using elastic adhesive, leaving the components bare. Therefore, it was thought that the evaluators had the impression of confusion and/or complication and scored “clear/confusing” and/or “complicated/easy” lower. Furthermore, it was thought that the evaluators had the impression of confusion and/or complication in how to switch measurement functions (such as how many times to press a button to switch to the function they wanted to measure) and in the arrangement of the sensors. In the future, we aim to improve Medic Hand by applying printed circuits and flexible electronics to conceal the wiring and sensors so that wearers can use the device without being aware of the components. In addition, we modify the way the wearer switches between the functions of the device and the placement of the sensors so that they are intuitive to the user.

Once the questionnaires were completed, the evaluators were interviewed for the four questions mentioned previously (Experimental Protocol and Statistical Analysis section). Regardless of SUS and UEQ scores, the evaluators had concerns about whether the Medic Hand would be damaged when worn. They cited the bare circuit board and wiring as the reason. This indicates that the appearance of a wearable device is an important factor in determining its acceptability for use. In fact, the evaluators cited resistance to using products on a daily life that might be damaged. On other hands, the evaluators also indicated that the novelty of Medic Hand lies in the fact that it can measure multiple biometric parameters through a single wearable device regardless of the SUS and UEQ scores (Question 4). This result is consistent with the scores from the UEQ scale (“attractiveness,” “stimulation,” and “novelty”), which indicated an Excellent rating. However, people who perceive themselves to be healthy are not interested in using wearable devices to measure biometric data on a daily basis and may not be receptive to their use. Therefore, to ensure that Medic Hand can be used and accepted widely, it is necessary to not only improve its usability but also build features such as an automatic triage decision-making system that will be advantageous to users^8–11^. Once it is understood that the public will benefit from the ability to collect biometric data efficiently with wearable devices, our goal of using the Medic Hand at disaster sites will be accepted.

In conclusion, we developed Medic Hand as a multi-biometric device that can measure the CRT, heart rate and oxygen saturation, skin temperature, and ambient temperature. The usability of Medic Hand was also evaluated on 16 nonmedical adults in their 20 s who majored in medical engineering, and both SUS and UEQ were scored to have above-average usability. As the results, The current features of the Medic Hand, which can efficiently measure biometric data when worn on the hand, were shown to have a positive effect on public acceptance, while the design assuming a male hand, exposed wiring and electronic boards, and non-intuitive switching of measurement functions were shown to have a negative effect. Therefore, in order for the Medic Hand to be accepted by the public, it is necessary to accommodate individual differences in the hand skeleton, to design an appearance that does not cause damage when worn, and to develop a system that utilizes the Medic Hand in order to understand the benefits of efficiently collecting multiple biometric data.

This study has the limitation that the evaluators were nonmedical adults. Cheng et al.^15^ stated that public understanding is necessary for acceptance of wearable devices at disaster sites, and in this study, nonmedical adults were selected as evaluators to identify factors that may or may not facilitate public acceptance of the Medic Hand. In this case, the evaluations may be biased in terms of such as “pragmatic quality, “ “attractiveness,” “stimulation,” and “novelty” depending on whether the evaluator is familiar with conventional devices for measuring biometric data. However, since the evaluators in this study were nonmedical adults majored in medical engineering, the bias was reduced because they have understood the measurement principles and operation of conventional devices for measuring biometric data. Furthermore, the SUS and UEQ only evaluate user experience and cannot evaluate the technical reliability of the Medic Hand. A high user experience during use is essential for the public acceptance of the Medic Hand^22^. The user experience of wearable devices is commonly evaluated by questionnaires such as SUS and UEQ^19^, and the evaluation method selected for this study is appropriate. We evaluated the technical reliability of the Medic Hand for measuring CRT in terms of agreement of measurements, reproducibility, and intra-rater reliability in a preliminary study^18^. However, the technical reliability of the pulse rate, oxygen saturation, and skin and environmental temperature measurements depends on the specifications of the sensors used. Therefore, we will study nurses and paramedics to evaluate the usability and technology reliability based on their working experience in the medical field. At the same time, considering that the Medic Hand is a system that is used under different weather, temperature, and lighting conditions, usability will be evaluated in the hospital, in ambulances, and outdoors, where triage is performed. In addition, we aim to conduct more accurate usability evaluations by expanding the age range for which the questionnaires are collected. The Medic Hand developed in this study is designed to assess “perfusion” as per the START method efficiently, so it cannot measure the respiratory rate. However, Medic Hand is equipped with a thermo-camera, and there are studies that have reported estimation of respiratory rate from images captured with a thermo-camera^23^. We will conduct another study to estimate the respiratory rate of a subject from thermographic images captured by Medic Hand so as to efficiently collect the biometric information necessary for evaluation based on the START triage method.

## Data Availability

The datasets generated and/or analyzed during the current study are available from the corresponding author upon reasonable request.
